# Supplementary material for: MetaRibo-Seq measures translation in microbiomes
Source: Nat Commun. 2020 Jun 29;11:3268. doi: 10.1038/s41467-020-17081-z (PMC7324362; doi:10.1038/s41467-020-17081-z)
Supplement: Supplementary file 10 — Supplementary Data 7 [file 41467_2020_17081_MOESM10_ESM.zip › File2/Confidence_High_Taxonomy/35005_out.krona.html]

Javascript must be enabled to view this page.

members
magnitude
magnitudeUnassigned
count
unassigned
taxon
rank

35005\_out

2

2
superkingdom
2

1239
phylum
2

2
class
186801

order
186802
2

2
31979
family

2
genus
1485

1896987

SRS015663\_contig\_number\_27412SRS051031\_contig\_number\_23879
species
2
